# Characteristics and Expression Patterns of the Aldehyde Dehydrogenase (ALDH) Gene Superfamily of Foxtail Millet (*Setaria italica L.*)

**DOI:** 10.1371/journal.pone.0101136

**Published:** 2014-07-02

**Authors:** Chen Zhu, Chen Ming, Xu Zhao-shi, Li Lian-cheng, Chen Xue-ping, Ma You-zhi

**Affiliations:** 1 Department of Chemistry, University of Science and Technology of China, Hefei, Anhui, China; 2 Institute of Crop Science, Chinese Academy of Agricultural Sciences, National Key Facility for Crop Gene Resources and Genetic Improvement, Key Laboratory of Biology and Genetic Improvement of Triticeae Crops, Ministry of Agriculture, Beijing, China; National Institute of Plant Genome Research, India

## Abstract

Recent genomic sequencing of the foxtail millet, an abiotic, stress-tolerant crop, has provided a great opportunity for novel gene discovery and functional analysis of this popularly-grown grass. However, few stress-mediated gene families have been studied. Aldehyde dehydrogenases (*ALDHs*) comprise a gene superfamily encoding NAD (P) ^+^-dependent enzymes that play the role of “aldehyde scavengers”, which indirectly detoxify cellular ROS and reduce the effect of lipid peroxidation meditated cellular toxicity under various environmental stresses. In the current paper, we identified a total of 20 *ALDH* genes in the foxtail millet genome using a homology search and a phylogenetic analysis and grouped them into ten distinct families based on their amino acid sequence identity. Furthermore, evolutionary analysis of foxtail millet reveals that both tandem and segmental duplication contributed significantly to the expansion of its *ALDH* genes. The exon-intron structures of members of the same family in foxtail millet or the orthologous genes in rice display highly diverse distributions of their exonic and intronic regions. Also, synteny analysis shows that the majority of foxtail millet and rice *ALDH* gene homologs exist in the syntenic blocks between the two, implying that these *ALDH* genes arose before the divergence of cereals. Semi-quantitative and real-time quantitative PCR data reveals that a few Si*ALDH* genes are expressed in an organ-specific manner and that the expression of a number of foxtail millet *ALDH* genes, such as, *SiALDH7B1*, *SiALDH12A1* and *SiALDH18B2* are up-regulated by osmotic stress, cold, H_2_O_2_, and phytohormone abscisic acid (ABA). Furthermore, the transformation of *SiALDH2B2*, *SiALDH10A2*, *SiALDH5F1*, *SiALDH22A1*, and *SiALDH3E2* into Escherichia coli (*E.coli*) was able to improve their salt tolerance. Taken together, our results show that genome-wide identification characteristics and expression analyses provide unique opportunities for assessing the functional roles of foxtail millet *ALDH* genes in stress responses.

## Introduction

As they grow, plants often encounter a wide spectrum of environmental stresses [1], such as drought, salinity, extreme temperatures and oxidative stress. To assure the survival and prosperity of their offspring, plants regulate the expression of a wide range of stress-responsive genes capable of coping with various abiotic stresses, which negatively affect plants by eliciting rapid and excessive accumulation of reactive oxygen species (ROS) that leads to cellular injury (e.g., lipid peroxidation or protein and nucleic acid modification) [2], [3]. Moreover, ROS reacting with lipids and proteins are known to cause an accumulation of toxic products (i.e. aldehydes), which in turn amplify ROS-induced damage. As such, a better understanding of the mechanisms involved in the evolutionary survival of plants to environmental stresses would be of great value.

Aldehyde dehydrogenases (ALDHs) represent an evolutionary conserved gene superfamily encoding NAD (P)^+^-dependent enzymes that catalyze the irreversible oxidation of a wide range of endogenous and exogenous aromatic and aliphatic aldehydes into corresponding carboxylic acids [4]. Several studies show that many ALDHs protect against various environmental stressors by indirectly detoxifying cellular ROS and/or reducing lipid peroxidation [5]. For instance, overexpression of AtALDH3 reduces lipid peroxidation and increases resistance to osmotic stress, metal toxicity, H_2_O_2_ and paraquat treatment [6]. Furthermore, ectopic expression of ALDH7 in both *Arabidopsis* and tobacco enhances their protection against various osmotic stressors, such as dehydration and high salinity [7]. In addition, overexpression of ALDH22A1 in transgenic tobacco plants increases its stress tolerance and leads to decreased levels of malondialdehyde (MDA) [8]. *ALDH* genes also play vital roles in many fundamental metabolic pathways under normal conditions. For example, they facilitate in the synthesis and catabolism of a wide of range of biomolecules, such as amino acids, lipids, and vitamins. As such, the ability of ALDHs to facilitate stress responses in plants has made these enzymes the focus of numerous studies on developing stress-resistant crops.

Aldehyde dehydrogenases constitute a diverse protein family found in various organisms. In 1999, the ALDH Gene Nomenclature Committee (AGNC) established criteria for cataloguing deduced ALDH protein sequences [9]. Sequences with more than 40% identity to a previously identified ALDH sequence represent a family and sequences with more than 60% identity within the ALDH family represent a protein subfamily. To date, ALDHs have been classified into 24 distinct families; *ALDH10*, *ALDH12*, *ALDH21*, *ALDH22*, *ALDH23*, and *ALDH24* are unique to plants [10]–[13]. Completion of the genomic sequencing of various plants has allowed more and more aldehyde dehydrogenase genes to be identified and classified from lower to higher plants, such as *Physcomitrella patens*, *Chlamydomonas reinhardtii*, *Arabidopsis thaliana*, *Vitis vinifera*, *Zea mays*, *Sorghum bicolor* and *Glycine max* [10], [12]–[15]. However, there have been any systematic investigations of aldehyde dehydrogenase families from the foxtail millet published.

Foxtail millet (*Setaria italica* L.) is well known for its drought tolerance and is one of the oldest cultivated millet crops and an important food and fodder grain crop in arid and semi-arid regions of Asia and Africa. It is a diploid C4 panicoid crop with a small genome of ∼515 Mb, a short life cycle, and a highly conserved genome structure relative to ancestral grass lineages [16]. As such, foxtail millet has been proposed as an ideal model crop for genetic and molecular studies. Recently, the US Department of Energy Joint Genomic Institute [17] and Beijing Genomics Institute (BGI), China [16] was able to fully sequence its genome, paving the way to additional genome-wide identification studies and the analysis of gene families in foxtail millet.

The SiNAC [18] and SiWD40 [19] gene families have recently been systematically analyzed. However, to date there are no reports on the analysis of the *SiALDH* gene family. In the current study, we identified 20 *ALDH* genes from foxtail millet, classified them into 10 different gene families, and investigated their expansion and evolutionary history by examining their duplication, chromosomal distribution, exon-intron structure and synteny map with their rice orthologs. Subsequently, we analyzed the expression profiles of these *SiALDH* genes in different tissues and under various abiotic stressors and identified the function of ten *SiALDH* genes expressed in transgenic *E.coli* in response to salinity-induced stress.

## Results and Discussion

### The foxtail millet ALDH gene family: nomenclature and phylogenetic analysis

Keywords, HMM profile, and BLAST searches uncovered that approximately twenty ALDH proteins exist in the *S.italica* genome. Furthermore, based on criteria established by the AGNC, 20 putative *ALDH* genes were also identified in *S.italica* genome and grouped into ten families (Table 1). Four families contained multiple members (*ALDH2*, six members; *ALDH3*, four members; *ALDH10* and *ALDH18*, two members each), each of the other six families was represented by a single gene (*ALDH5, ALDH6, ALDH7, ALDH11, ALDH 12*, and *ALDH22*) and each gene was assigned to different subfamilies (Table 1). A unique identifier was assigned to each of the *S.italica* ALDH proteins. A number, based on the present location of the *ALDH* gene on the chromosome, following the subfamily name was used to distinguish multiple members contained in a single family. Subcellular localization predictions revealed that most of *S.italica* ALDH proteins are located in the cytoplasm and mitochondrion. Moreover, ten *S.italica* ALDH proteins had splice variant and only primary transcripts were selected for phylogenetic and comparative analyses. Among the 20 foxtail millet *ALDH* genes identified, eighteen genes were supported by full length protein sequences and cDNA sequences (their corresponding GeneBank Accession are listed in Table 1); the other two genes were not found in GeneBank, but the full length sequences are shown in the Text S1.

**Table 1:** Foxtail millet ALDH genes and superfamilies.

Family	Gene Locus ID	Annotation	Accession No.	CDS (bp)	ORF (aa)	Subcellular Localization
**Family 2**	Si000898m	SiALDH2C2	XM_004968994	1662	553	Cytoplasm
	Si000743m	SiALDH2C3	XM_004968990	1818	605	Mitochondrion
	Si040073m	SiALDH2C4	No entry	1359	453	Peroxisome
	Si006255m	SiALDH2C1	XM_004965940	1545	514	Cytoplasm
	Si006183m	SiALDH2B2	XM_004965148	1650	549	Mitochondrion
	Si016807m	SiALDH2B1	XM_004953741	1668	555	Mitochondrion
**Family 3**	Si009981m	SiALDH3H1	XM_004977167	1449	482	Cytoplasm
	Si026307m	SiALDH3H2	No entry	1434	447	Cytoplasm
	Si009984m	SiALDH3E2	XM_004976343	1446	481	Cytoplasm
	Si017050m	SiALDH3E1	XM_004953227	1458	485	Cytoplasm
**Family 5**	Si016884m	SiALDH5F1	XM_004951692	1587	528	Mitochondrion
**Family 6**	Si029327m	SiALDH6B1	XM_004955659	1746	581	Cytoplasm
**Family 7**	Si029116m	SiALDH7B1	XM_004956875	2055	684	Mitochondrion
**Family 10**	Si009902m	SiALDH10A2	XM_004975822	1518	505	Mitochondrion
	Si013592m	SiALDH10A1	XM_004973405	1518	505	Cytoplasm
**Family 11**	Si013613m	SiALDH11A1	XM_004973492	1497	498	Cytoplasm
**Family 12**	Si021508m	SiALDH12A1	XM_004961322	1833	610	Cytoplasm
**Family 18**	Si000348m	SiALDH18B2	XM_004970516	2412	803	Cytoplasm
	Si021235m	SiALDH18B1	XM_004961829	2457	818	Cytoplasm
**Family 22**	Si029482m	SiALDH22A1	XM_004958707	1581	526	Cytoplasm

In the present study, we listed the numbers of gene family members for each individual ALDH family in *S.italica* and ten other plant species (*Arabidopsis thaliana, Oryza sativa, Sorghum bicolor, Zea mays, Vitis vinifera, Populus trichocarpa, Physcomitrella patens, Chlamydomonas reinhardtii, Volvox carteri*, and *Glycine max*) and *Homo sapiens* (Table S1). Of note, there is a lack of ALDH1 and ALDH4 gene family members found in plants due to nomenclature errors made when the genes were originally identified [12]. Plants have 13 various ALDH gene families: *ALDH2*, *ALDH3*, *ALDH5*, *ALDH6*, *ALDH7*, *ALDH10*, *ALDH11*, *ALDH12*, *ALDH18*, *ALDH21*, *ALDH22, ALDH23*, and *ALDH24*; in addition, *ALDH19* has only been identified within tomato genome [20]. ALDH21, ALDH23 and ALDH24 are unique to lower plants. Like other monocot/dicot plants, *S.italica* contain ten common core ALDH families: *ALDH2*, *ALDH3*, *ALDH5*, *ALDH6*, *ALDH7*, *ALDH10*, *ALDH11*, *ALDH12*, *ALDH18*, and *ALDH22*). Unfortunately, the evolutionary relationships of the ALDH protein in foxtail millet and other plants had never been performed.

**Figure 1:**
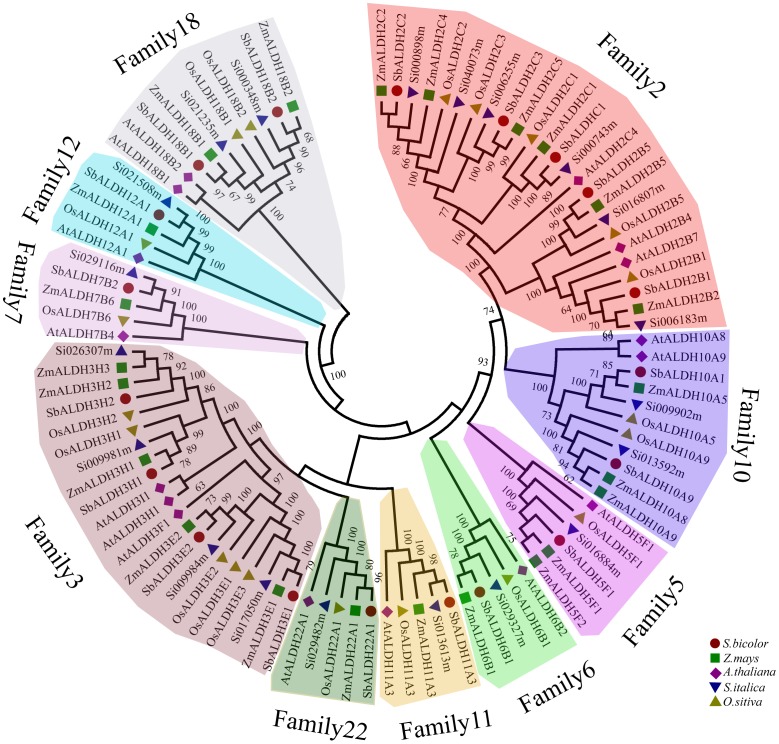
Phylogenetic analysis of foxtail millet and other plant *ALDHs*. Phylogenetic tree was constructed with *ALDH* protein sequences from *S.bicolor* (Sb), *Z.mays* (Zm), *O.sativa* (Os), *S.italica* (Si), and *A. thaliana* (At). Members of respective ALDH families are depicted in a specific background color.

In recent years, the ALDH protein in multiple plant species has been identified. As such, we chose several representational plants for analysis, including *Arabidopsis thaliana, Oryza sativa, Sorghum bicolor, and Zea mays* and constructed a phylogenetic tree of the *ALDH* genes in these species, in addition to foxtail millet. The tree was classified into ten major families (Fig. 1 & Table 1) and ALDH proteins from the same families were clustered together (Fig. 1). The phylogenetic tree also revealed that these plant ALDHs split into three clades and share the common core plant ALDH families, listed above. In addition, we found that the majority of foxtail millet *ALDH* families are more closely related to those in grass species (*O. sativa*, *Z. Mays*, *and S. Bicolor*). These results are consistent with the present understanding of plant evolutionary history [21].

### Segmental and tandem duplication contribute to superfamily expansion

**Figure 2:**
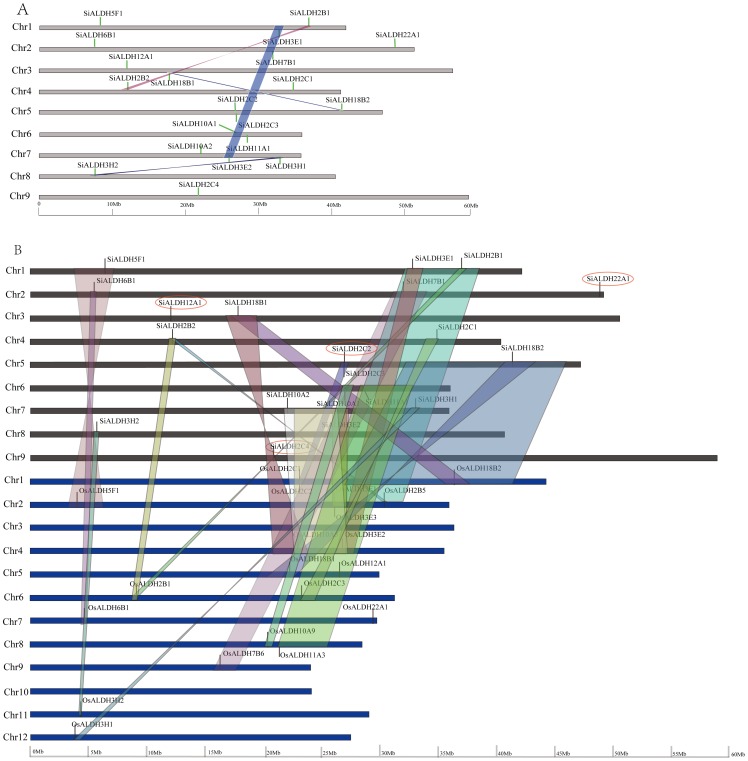
Distribution and synteny of ALDH genes on foxtail millet chromosomes and synteny analysis of ALDH genes between foxtail millet and rice. (A) Chromosomes 1–9 are depicted as horizontal gray bars. ALDH genes are indicated by vertical green line. Colored bars denote syntenic of the foxtail millet genome. (B) Foxtail millet and rice chromosomes are depicted as horizontal black and blue bars, respectively. Foxtail millet and rice ALDH gene are indicated by vertical black lines, Colored bars denote syntenic regions between foxtail millet and rice chromosomes. A twisted colored bar indicates that syntenic regions are in opposite orientations. SiALDH genes don't located in the syntenc blocks between rice and foxtail millet chromosomes were marked by red oval.

Gene duplication plays an important role in the expansion of gene families. It was recently reported that the foxtail millet genome underwent whole-genome duplication, similar to other grasses, approximately 70 MYA (million year ago) and most of the duplications were generated in the whole genome duplication (WGD) event shared by all grasses [16]. In the present analysis, we found that the 20 foxtail millet *ALDH* genes are randomly distributed on nine foxtail millet chromosomes (Fig. 2A). Among them, eight genes (*SiALDH3E1*/*SiALDH3E*2, *SiALDH3H1*/*SiALDH3H2*, *SiALDH2B1*/*SiALDH2B2* and *SiALDH18B1*/*SiALDH18B2*) are located in four pairs of segmental duplicated genome regions, likely caused by whole genome duplication. In addition, *SiALDH2C*2 and *SiALDH2C3* genes are identified as a tandem duplication in foxtail millet ALDH family. In summary, four foxtail millet multi-member *ALDH* gene families are linked with either segmental or tandem duplication events, suggesting that segmental and tandem duplication events play significant roles in the expansion of *SiALDH* genes. Moreover, the contribution of selection on coding sequences can be quantified by measuring the ratio of nonsynonymous to synonymous substitutions (Ka/Ks). A pair of sequences with Ka/Ks<1 indicates that one sequence has undergone purifying selection while the other has been drifting neutrally. Alternatively, if Ka/Ks = 1 then both sequences have been drifting neutrally. And finally, in the rare event that Ka/Ks>1 specific sites in that sequence have been under positive selection [22]. Our results indicate that the Ka/Ks ratios for five duplicated pairs varied from 0.0024 to 0.1726 with an average of 0.06998 (Table S2), strongly indicating that the SiALDH family underwent strong purifying selection pressure. All five paralogous pairs showed Ks values >1.11, signifying that these duplications might have occurred >85.48 MYA (Table S2). This result suggests that duplications of these 5 paralogous pairs occurred before the whole genome duplication (WGD) of grass. However, we are cautions about asserting that theses *SiALDH* duplicated before the WGD, considering that the pseudogenization or frame-shift mutation have the potential to inflate Ks value [23].

Structural divergences are prevalent in duplicate genes and, in most cases, have led to the generation of functionally distinct paralogs [24]. Investigation into the gene structures of multiple gene families (Fig. S1) shows that duplicate genes of *SiALDH* have a highly diverse distribution of exons and introns in the foxtail millet genome. In addition, nonduplicate genes of *SiALDH* also have different gene structures. For example, *ALDH10* genes from rice [25] and grape [12] had the same numbers of exons while exhibiting nearly identical lengths, whereas the first exon of *SiALDH10A1* and *SiALDH10A2* was clearly different in the foxtail millet genome. We hypothesize that this difference is attributed to a rapid evolution of these genes through gene duplications or via their integration into the genome following reverse transcription [26]–[28].

### Evolutionary relationship of *ALDH* gene families between foxtail millet and rice

Researchers can gain a better understanding of the genomic structure/evolution of a lesser-studied taxon via comparative genomic analyses [29]. As such, we were able to infer the probable function of the orthologous genes by comparing the genomes of foxtail millet and rice. Large-scale synteny analysis identified 90% (18/20) of *SiALDH* genes to have shared synteny with their orthologs of the rice genome. Among them, 10 pairs (*SiALDH2C3/OsALDH2C1*, *SiALDH2C1/OsALDH2C3*, *SiALDH3H1/OsALDH3H1, SiALDH3H2/OsALDH3H2*, *SiALDH5F1/OsALDH5F1*, *SiALDH6B1/OsALDH6B1*, *SiALDH7B1/OsALDH7B6*, *SiALDH10A2/OsALDH10A5*, *SiALDH10A1/OsALDH10A9*, and *SiALDH11A1/OsALDH11A3*) unambiguously existed in both foxtail millet and rice genomes. There were some much stronger cases for a gene/regional duplication where two separate genes of foxtail millet were related to one or two genes in the rice genome, such as *SiALDH2B1/SiALDH2B2-OsALDH2B5*, *SiALDH3E1/SiALDH3E2-OsALDH3E1* and *SiALDH18B1/SiALDH18B2-OsALDH18B1/OsALDH18B2*. These results suggest that the majority of *SiALDH* genes share a common ancestor with *OsALDH* genes counterparts. Certainly, some *SiALDH* genes were not mapped to any syntenic blocks with rice, such as *SiALDH2C2*, *SiALDH2C4*, *SiALDH12A1*, *and SiALDH22A1* (Figure 2B), but it does not mean that these ALDH genes from foxtail millet and rice do not share a common ancestor. Rather, this can be explained by the fact that foxtail millet and rice chromosomes have undergone extensive rearrangements and fusions that possibly lead to selective gene loss [16].

We next want to determine if functional conservations have been maintained between orthologous genes in the two related species. As such, we calculated the ratios of nonsynonymous (Ka) to synonymous (Ks) substitution rate (Ka/Ks) for orthogous gene pairs of foxtail millet *SiALDHs* with those of rice (22 pairs). We found that the ratios of Ka/Ks for all 22 orthologous gene pairs were less than 1, indicating that functional conservation is likely maintained between these orthologous pairs. We further estimated the approximate divergence time of those orthologous gene pairs. Eight orthologous gene pairs (*SiALDH2B2-OsALDH2B1, SiALDH5F1-OsALDH5F1*, *SiALDH6B1-OsALDH6B1*, *SiALDH7B1-OsALDH7B6*, *SiALDH10A1-OsALDH10A9*, *SiALDH10A2-OsALDH10A9*, *SiALDH11A1-OsALDH11A3*, and *SiALDH18B1-OsALDH18B1*) have calculated Ks values that vary from 0.3481 to 0.5655 with an average of 0.4467, indicating that the divergence time of these genes was somewhere between 26.77 and 43.50 MYA (Table S2) at a period of time following the divergence between Potidaea and Panicoideae. Additionally, five of the orthologous pairs (*SiALDH2C1-OsALDH2C3*, *SiALDH2B1-OsALDH2B5*, *SiALDH3H1-OsALDH3H1*, *SiALDH3H2-OsALDH3H2*, *SiALDH18B2-OsALDH18B2*) had Ks values from 0.6273 to 0.8862 with an average of 0.7487 (Table S2), signifying that their divergence occurred at some point between 48.25 and 68.17 MYA, after the whole genome duplication (WGD) of grass. Taken together, these results are comparable to evolutionary studies of protein-coding genes annotated from a recently released draft genome sequence of foxtail millet. Alternatively, the other nine orthologous pairs showed Ks values larger than 1 (Table S2), demonstrating that these orthologous pairs occurred before he whole genome duplication (WGD) of grass, but we also are cautions about these results and believe that further research is required to be certain of this data.

**Figure 3:**
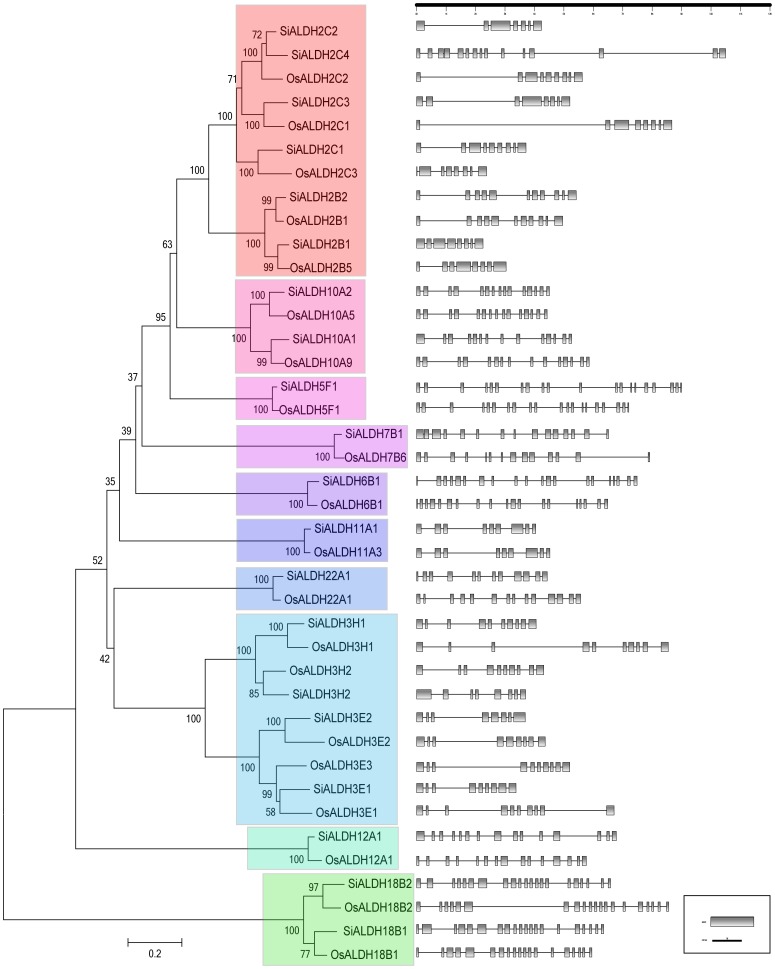
Phylogenetic analysis and exon-intron structures of foxtail millet and rice ALDH genes. Numbers above or below branches of the tree indicate bootstrap values. Coding exons, represented by ashy, were drawn to scale. Dashed lines connecting two exons represent introns. Members of respective *ALDH* families are depicted in a specific background color.

Orthologous genes conceivably had identical functions, but tended to diverge in regulatory and coding regions which led them to altered the expression patterns and acquire new functions, respectively [24]. In this study, we compared the exon-intron structures of *ALDH* genes identified in the foxtail millet genome with those found in rice. Our results revealed that a number of exonic losses and gains occurred during the evolution of *ALDH* genes in both species at the 5′ end or 3′ terminal and even in the middle of their sequences, such as *OsALDH18B1*/*SiALDH18B1*, *OsALDH2B5*/*SiALDH2B1* (Fig. 3). Furthermore, *SiALDH18B1* have acquired an additional exon between the first and second exon of *OsALDH18B1* (Fig. 3), *OsALDH2B5* and *SiALDH2B1* have the same number of exons but exhibit different exons lengths (Fig. 3), suggesting that these foxtail millet *ALDH* genes might possess different functions. On the other hand, compared to rice, several *SiALDH* genes have identical exon/intron structure (*OsALDH5F1*/*SiALDH5F*1, *OsALDH11A3*/*SiALDH11A*1, *OsALDH10A5*/*SiALDH10A*2, *OsALDH2B1*/*SiALDH2B2*, and *OsALDH3H1*/*SiALDH3H1*) (Fig. 3), indicating that a portion of foxtail millet *ALDH* genes have similar gene functions to *ALDH* genes in rice. However, the insertion and deletion of amino acids in the proteins with identical gene structures should not to be disregarded. As such, further investigations are essential in order to illustrate the specifics of any functional divergence between *SiALDH* genes.

### Expression profiles and promoter analysis of foxtail millet *ALDH* genes

**Figure 4:**
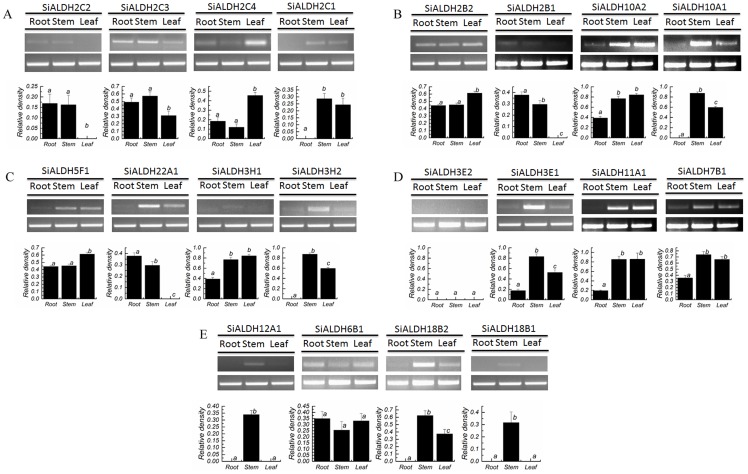
Semi-quantitative RT-PCR results of 20 foxtail millet ALDH genes in root, stem, and leaf. (A), (B), (C), (D) and (E): The RT-PCR products, generated with 20 SiALDHs and actin (AF288226.1) gene specific primers, were electrophoresed in a 1.5%agarose gel and densitometric analysis of the corresponding band to SiALDHs. The bars represent the mean ± SD of the results from three separate experiments. One-way ANOVA analysis of variance showed significant differences between group means (P<0.05). Tukey's multiple comparison test showed significant differences between the means of groups depicted by the different letters on the bars (P<0.05). Actin mRNA (AF288226.1) was used as an internal control.

We ran semi-quantitative RT-PCRs to observe the organ-specificity of *SiALDH* genes using three tissues: young root, stem, and leaf. We found that the majority of *SiALDH* genes were expressed in the stem and leaf and had significantly lower expression levels (and in some cases were even were non-detectable) in the root, such as *SiALDH7B1*, *SiALDH2C1*, *SiALDH5F1*, and *SiALDH22A1*. *SiALDH12A1* and *SiALDH18B1* were mainly expressed in the stem and *SiALDH3E1* was expressed in the root, stem and leaf, unlike its sister gene, *SiALDH3E2*, which was not expressed in the three tissues. Similar results were observed in other duplicated pairs, such as *SiALDH18B1*/*SiALDH18B2* and *SiALDH2C2*/*SiALDH2C3* (Fig. 4).

A comprehensive promoter analysis is capable of providing a reference for functional predictions of the 20 stress-related foxtail millet ALDH genes. For this purpose, we identified regulatory elements were identified in the DNA sequences (∼1 kb region upstream of the predicted *SiALDH* genes) using plantCARE [30]. A number of regulatory elements, including MBS, ARE, LTR and ABRE, known to be involved in responses to either abiotic stresses or hormone levels were found to be overrepresented in the 1kb region upstream of the *SiALDH* gene (Table S3). The 1 kb region upstream of all *SiALDH* genes have light or ABA regulatory elements (Table S3). Taken together, this data may indicate that the predicted *SiALDH* genes have a crucial role in stress-mitigation in the foxtail millet.

**Figure 5:**
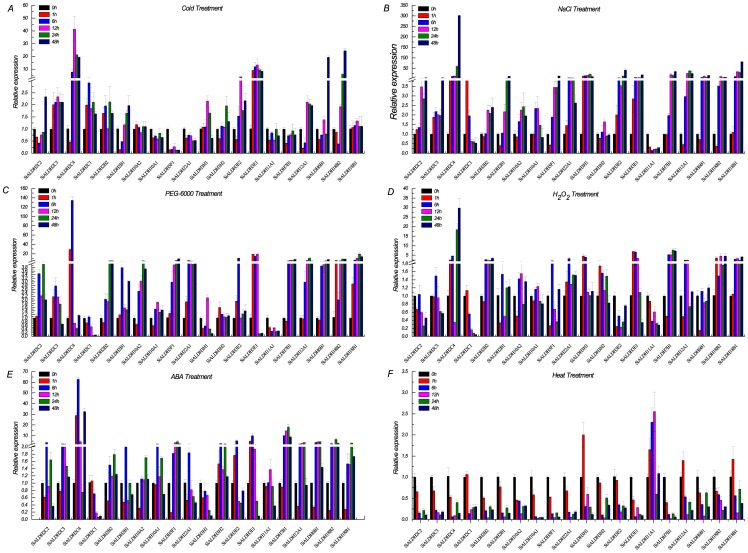
QRT-PCR analysis of the 20 foxtail millet *ALDH* genes. Time course expression analysis of the 20 foxtail millet genes under various stresses. (A) Cold at 4°C; (B) 250 mM NaCl; (C) 20%PEG-6000; (D) 200 µM H_2_O_2_; (E) 100 µM ABA; (F) Heat at 42°C. Actin mRNA (AF288226.1) was used as an internal control. The bars represent the mean± SD of the results from three separate experiments.

We next subjected the 20 SiALDH genes to quantitative expression analysis in order to decipher the role of SiALDH genes during diverse environmental situations: dehydration (20% PEG-6000), salinity, high temperature, cold, ABA, and H_2_O_2_ stress (0, 1, 6, 12, 24, 48 h treatment durations) (Fig. 5 and Table S4). *ALDH2* family members required for male plant fertility were first identified in Maize [31], [32]. Soon afterwards, a number of publications showed that plant *ALDH* family members are associated with cell wall strength [33], [34], aluminum stress [35], and protection against pathogen infection [36]. In the current paper, we found that *SiALDH2* genes are induced by abiotic stressors and hormones. Namely, under PEG-6000 and ABA treatment, all of the *SiALDH2* genes were up-regulated at least one time points, except for the expression of *SiALDH2C1*, which was down-regulated under ABA treatment (Fig. 5C and E). Following cold and NaCl treatment, the expression levels of the all *SiALDH2* genes increased at a number of time points and after 6 h (Fig. 5A and B). H_2_O_2_ treatment, the expression profiles of *SiALDH2C4* and *SiALDH2B2* both rapidly increased at 1h and 6h post-treatment, respectively, and remained elevated throughout the experiment, while the expression of *SiALDH2C2* and *SiALDH2C1* decreased or remained at baseline (Fig. 5D). The expression levels of the all other *SiALDH2* genes were slightly increased. Following heat treatment, the transcript levels of *SiALDH2* genes were down-regulated (Fig. 5F).

*ALDH3, ALDH7, ALDH10, ALDH11, ALDH12, and ALDH18* gene families contain several stress inducible *ALDH* genes that have been identified in various plants. Many *ALDH3* genes are believed to be regulated by the ABA stress-response pathway. For example, in Arabidopsis, *AtALDH3I1* is induced by ABA exposure, salinity, dehydration, heavy metals, oxidants, and pesticides [6], [37], [38] and *AtALDH3H1* also known to be stress-responsive. In this study, we were able to induce *SiALDH3H1* via H_2_O_2_, cold, and NaCl (Fig. 5A, B and D) whereas the expression levels of *SiALDH3H2* displayed no significant difference under NaCl and PEG-6000 treatment (Fig. 5B and C). Furthermore, the transcript levels of *SiALDH3E2* and *SiALDH3E1* were up-regulated under various stressors, with the exception of *SiALDH3E2* following H_2_O_2_ and heat treatment, which was down-regulated (Fig. 5A-F), while the expression of *ALDH3E* genes in rice was down-regulated under both drought and high salinity stresses [25].

A previous study established that ectopic expression of *ALDH7* in both Arabidopsis and tobacco enhances their tolerance to drought, salinity and oxidative stress [7]. Rice *ALDH7* can also be induced by oxidative and abiotic stresses [39]. Here, we found that *SiALDH7B1*, which has the closest phylogenetic relationship with *OsALDH7*, was induced by all various stressors except for low temperature. Moreover, the *ALDH10* family, encoding for betaine aldehyde dehydrognases (BADHs; EC 1.2.1.8), are known to participate in the generation of osmolyte and the quaternary ammonium compound glycine betaine [40], [41]. Glycine betaine (GB) is a non-toxic cellular osmolyte that plays a key role during hyperosmolyte conditions and helps to stabilize the protein structure and to maintain the integrity of membranes against the damaging effects of excessive abiotic stresses [42]. In the current research, both *SiALDH10A1* and *SiALDH10A2* were induced by PEG-6000, NaCl, H_2_O_2_ and ABA (Fig. 5B-E). However, neither of the genes responded to cold treatment (Fig. 5A).

The *ALDH11* gene family, encodes a cytosolic glyceraldehyde-3-phosphate dehydrogenase that catalyzes the irreversible NADP^+^-dependent oxidation of GAP to 3-phosphoglycerate and NADPH [43]. The protein plays a role in the response to desiccation in *A. thaliana* and *Craterostigma plantagineum* [44], [45]. Likewise, this gene can also be induced by other environmental stress conditions, such as heat shock and anaerobic stress. Here we found that the expression of *SiALDH11A1* was down-regulated under cold, PEG-6000, H_2_O_2_, and salt stresses (Fig. 5B–E), similar results were previously published on rice under drought stress [25]. Furthermore, heat stress actually increased the expression levels of *SiALDH11A1* (Fig. 5F), suggesting potential roles for this gene in mediating cell damage during heat shock.

In plants, *ALDH12* family members play a key role in proline (Pro) degradation and their expression levels have been shown to be up-regulated via various stresses [46]. For example, both exogenous proline and salinity can induce the expression levels of *AtALDH12A1*. Moreover, *OsALDH12* gene is induced by drought and salt stresses. Our data (**Fig. 5A–F**) reveals that *SiALDH12* was upregulated under all abiotic stress conditions and ABA treatment, suggesting a possible role for it in foxtail millet during periods of environmental hardship.

The Si*ALDH18* family, encoding a Δ-1-pyrroline-5-carboxylate synthetases (P5CSs; EC 1.2.1.41 and EC 2.7.2.11), is a bifunctional protein that contains an N-terminal amino-acid kinase domain and a C-terminal aldehyde dehydrogenase domain. These enzymes play an important role in the biosynthesis of proline [47]. Furthermore, the expression levels of *ALDH18* in a number of plants have been shown to be up-regulated in response to osmotic stress [48], [49]. Similarly, we also showed that *SiALDH18* was up-regulated following PEG-6000, NaCl, H_2_O_2_, low temperature, and ABA (**Fig. 5A–E**).

The *ALDH5* gene family is comprised of succinic semialdehyde dehydrogenases (SSADH; EC 1.2.1.24), which participates in GABA ‘shunt’ pathway in bacteria, plants and animals. In plants, *ALDH5* mutations cause enhanced accumulation of reactive oxygen intermediates and cell death in response to light and heat stress [50]. Our results (**Fig. 5A–F**) indicate an upregulation of *SiALDH5* by all of the investigated stressors except for low temperature.

The *ALDH6* gene family is composed of methylmalonyl semialdehyde dehydrogenases (EC 1.2.1.27). Thus far, the function of these genes in plants is not clear. Here we found an upregulation in the expression of Si*ALDH6B1* under PEG-6000, NaCl, and ABA treatment (**Fig.5** **B, C and E**). However, we believe their exact functions should be elucidated further. Moreover, *ZmALDH22A1* was recently found to be up-regulated in response to a variety of stressors such as dehydration, high salinity, and ABA treatment [51]; the same consequence in foxtail millet is displayed in **Fig. 5A–F**. Nonetheless, *ALDH22* is not known to be induced by osmotic stress in rice and *Arabidopsis* [25], [52]. As such, the role of *ALDH22* in plants still needs further study.

In summary, the overall variability in gene expression patterns implies that *SiALDHs* participate in a complex network of pathways in order to perform different physiological functions in response to different challenges. This comprehensive expression profile provides a clue to the role of *SiALDHs* in imparting stress tolerance.

### Enhancement of salt stress tolerance of recombinant *E.coli* harboring foxtail millet *ALDH* genes

The expression of foreign plant genes can directly contribute to increasing stress tolerance in bacteria host cells [53], [54]. In plants, abiotic stress can trigger the generation of reactive oxygen species (ROS) that disrupts cellular homeostasis and induce the expression of genes involved in defense mechanisms [55]. Moreover, aldehyde dehydrogenases play a key role in the detoxification of various aldehyde molecules produced in response to abiotic stress. Thus, we expressed ten SiALDH proteins (SiALDH2C2, SiALDH2C1, SiALDH2B2, SiALDH10A2, SiALDH5F1, SiALDH22A1, SiALDH3E1, SiALDH3E2, SiALDH11A1, and SiALDH12A1) in *E.coli* in an attempt to determine the function of foxtail millet ALDH proteins to salt stress condition, SDS-PAGE analysis results demonstrated that the molecular weights of the ten recombinant proteins agreed with the predicted molecular weights (Fig. S2). Subsequently, we spotted aliquots of recombinant strains harboring the empty vector pET-28a (Control) and 10 recombinant vectors (pET-SiALDH2C2, pET-SiALDH2C1, pET-SiALDH2B2, pET-SiALDH10A2, pET-SiALDH5F1, pET-SiALDH22A1, pET-SiALDH3E1, pET-SiALDH3E2, pET-SiALDH11A1, and pET-SiALDH12A1) onto LB plates and supplemented them with either 500 mmol/L or 800 mmol/L NaCl. The growth status of Rosetta cells (transformed with Control and recombinant vectors) indicated that the recombinants and control cells showed similar growth on LB medium, suggesting that exogenous SiALDHs do not restrain the cell growth.

**Figure 6:**
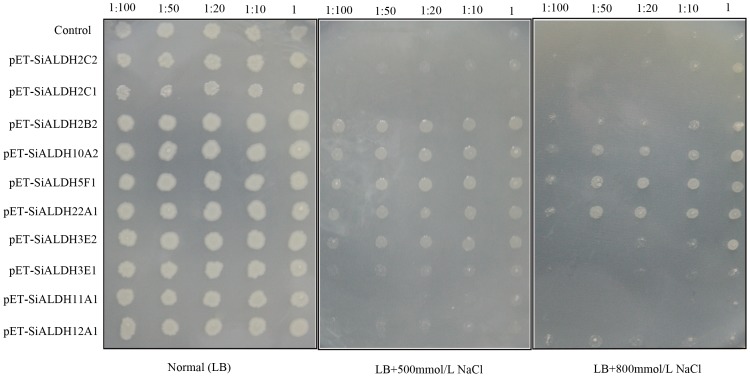
Spot assay of E. coli growth. The viabilities of the strains were detected on different LB plated with additional 500/L NaCl (B), 800 mmol/L NaCl and normal LB plates (A). Strains were Control: strain harboring plasmid pET-28(a), pET-ALDHs: strain harboring different SiALDH proteins. 1.5 microliters of the serially diluted E. coli cells cultured for 16 h at 16°C with 0.5 mmol/L IPTG were dotted onto LB agar plates containing NaCl at the marked concentration.

Results show that high salt severely inhibits the growth of the control strain. Amazingly, the five recombinants (pET-SiALDH2B2, pET-SiALDH10A2, pET-SiALDH5F1, pET-SiALDH22A1, and pET-SiALDH3E2) were able to grow normally at different dilution gradients under 500 mmol/L NaCl (Fig. 6). Similarly, under 800 mmol/L NaCl, we can clearly see that Rosetta cells harboring *SiALDH10A1*, *SiALDH22A1*, and *SiALDH5F1* were able to still grow normally at high bacterial concentrations (Fig. 6), revealing that these five *SiALDH* genes (*SiALDH2B2*, *SiALDH10A2*, *SiALDH5F1*, *SiALDH22A1*, and *SiALDH3E2*) are associated with high salt tolerance. This enhancement of salt tolerance further indicates that the expression of these foxtail millet *ALDH* proteins in host cells is able to confer their protective function against protein damage, cellular membrane disruption, and cellular apoptosis; their exact function in plants needs to be further examined.

### Summary

Aldehyde dehydrogenases (ALDHs) are a set of NAD (P)^+^-dependent enzymes that oxidize a wide range of aldehydes into their corresponding carboxylic acids. The identification and characterization of ALDH gene families has been well studied in different plant species. However, currently that are no reports investigating ALDH gene families in the foxtail millet. In the current study, we identified 20 ALDH genes in the foxtail millet genome, grouped them into ten families, and then assigned each a unique identifier to each of the predicted *S.italica* ALDH proteins based on the criteria established by the ALDH Gene Nomenclature Committee (AGNC). Importantly, evolutionary analysis reveals that both tandem and segmental duplications have contributed significantly to the expansion of the foxtail millet *ALDH* genes. Our gene structure analysis shows that *ALDHs* from the same family in foxtail millet or the orthologous genes in rice display highly diverse distributions of their exonic and intronic regions. Comparative synteny analysis between foxtail millet and rice genomes show that the majority of *SiALDH* and *OsALDH* gene homologs are located in syntenic regions between the two, indicating that these ALDH genes share common ancestors. In addition, we found that analysis of the Ka/Ks ratios of paralogous and orthologous gene pairs facilitates in a deeper understanding of the evolutionary significance of gene-duplications and divergence. Furthermore, we show for the first time that a few Si*ALDH* genes extremely organ-specific and that the expression pattern of a number of *SiALDH* genes is affected by several stressors, including dehydration, salinity, cold, ABA treatment, and heat stress. Finally, we find that the transformation of *SiALDH2B2*, *SiALDH10A2*, *SiALDH5F1*, *SiALDH22A1*, and *SiALDH3E1* into *E.coli* was able to improve its salt tolerance. Taken together, this work lays a solid foundation for future studies on the function of ALDH genes in foxtail millet and many other grasses, as well.

## Methods and Materials

### Identification and annotation of foxtail millet *ALDH* genes

Rice [15] and *A. thaliana* [10] *ALDH* amino acid sequences were retrieved from the PHYTOZOME v9.1 database (http://www.phytozome.org/) and utilized to identify homologous peptides from foxtail millet by BLASTP search using default settings. The keywords “ALDH”, “Aldehyde dehydrogenases”, and the HMM profiles of the ALDH domain PF00171, KOG2450 (aldehyde dehydrogenase), KOG2451 (aldehyde dehydrogenase), KOG2453 (aldehyde dehydrogenase) and KOG2456 (aldehyde dehydrogenase) were all used as queries for searching the foxtail millet genomic database to identify *ALDH* and *ALDH*-like sequences [56]. Similarity searches were also performed through BLASTP in GeneBank non-redundant protein database to eliminate possible exclusions of any additional ALDH member. All hits with the excepted values <1.0 were retrieved and all redundant sequences were removed using the decrease redundancy tool (http://web.expasy.org/decrease_redundancy/). Each non-redundant sequence was monitored for the presence of the conserved ALDH domain (PF00171) by SMART (http://smart.embl-heidelberg.de/) [57], Pfam (http://pfam.sanger.ac.uk/) and CDD (Conserved Domain Database) [58] searches. Likewise, the two active sites (PS00070 (cysteine active site) and PS00687 (glutamic acid active site)) were checked by PROSITE (http://prosite.expasy.org/). The deduced *ALDH* polypeptides were annotated using criteria established by the *ALDH* Gene Nomenclature Committee (AGNC) [3], [9]. In brief, deduced amino acid sequences greater than 40% identical to other previously identified *ALDH* sequences were defined as a family, while sequences with greater than 60% identity were defined as a protein subfamily. Deduced amino acid sequences with less than 40% identity described a new *ALDH* protein family. YLoc [59], [60] was used to predict the foxtail millet ALDH gene subcellular localization.

### Sequence alignments, phylogenetic and promoter analysis

The protein sequences of *ALDH* genes in *A. thaliana, O. sativa*, *S*. *Bicolor*, *Z*. *Mays* [10] and *S.italica* were also retrieved from the PHYTOZOME v9.1 database (http://www.phytozome.org/). Subsequently, we performed multiple alignments of *ALDH* protein sequences using ClustalX2.0 [61]. The alignments results was adjusted using BioEdit V7.0.5.3 [62] and eliminated the portions of the sequences that could not be reliably aligned. Phylogenetic trees were constructed with the MEGA5.10 Beta4 [63] software utilizing the neighbor-joining (NJ) method and the bootstrap test was replicated 1000 times. The entire alignment has been provided as Text S2.

In order to investigate the promoter regions of the *SiALDH* gene family, the 1 kb upstream regions (based on the position of the genes provided by the *S.italica* annotation information) were selected and analyzed using plantCARE [30]. Known as stress-mediated regulatory elements are listed in **Table S3**.

### Physical Mapping, Gene Duplication and Gene Structure Analysis

Specific chromosomal location and segment duplication of SiALDH genes were determined by FeatView (http://genomevolution.org/CoGe/FeatView.pl) tool. The genes were plotted separately on to all nine foxtail millet chromosomes according to their ascending orders of physical position (bp) and then displayed using Adobe Illustrator CS6. Duplications in the foxtail millet genome and synteny between foxtail millet and rice were established utilizing the SynMap tool (detailed settings are as following: Blast Algorithm: Last, DAGChainer options and Merge syntenic Blocks use the recommended parameters, and the synonymous (Ks) and non-synonymous (Ka) substitution rates were calculated using the CODEML program [64]) and confirmed with the GEvo tool at the CoGe Web site (http://synteny.cnr.berkeley.edu/CoGe/). The formulas T = Ks/2λ (λ = 6.5×10^−9^) [65], [66] was used to calculate time of duplication and divergence of each *SiALDH* genes. Next, we identified tandem duplications of *ALDH* genes in the foxtail millet genome by checking their physical locations in individual chromosomes. Tandem duplicated genes were defined as adjacent homologous *ALDH* genes on the foxtail millet chromosomes, with no more than one intervening gene. All paralogous and orthologous gene pairs are listed in **Table S5** and displayed using Adobe Illustrator CS6. Finally, The exon/intron structures of foxtail millet *ALDH* genes were determined from alignments of their coding sequences with corresponding genomic sequences using the est2genome program [67]. The diagrams of exon-intron structures were obtained using the online program FancyGene [68].

### Plant materials, growth conditions and treatments

The foxtail millet variety, Tie-8396, was used for all experiments. The seeds were germinated on wet filter paper at room temperature for 2 days and planted in pots with a mixture of peat/forest and vermiculite (1∶1 v/v). Plants were grown in a greenhouse (28°Cday/20°Cnight, 16 h photoperiod, natural lighting, 70% relative humidity) and when the seedlings were five weeks old they were chronologically plunged into a 250 mM NaCl solution, a 100 µM ABA solution, a 20%PEG-6000 solution, and 100 µM H_2_O_2_ solutions for multiple periods of time (0, 1, 6, 12, 24, and 48 h). Moreover, we also subjugated the seedlings to 4 h of 42°C heat stress in an incubator followed recovery (0, 1, 6, 12, 24, and 48 h) at room temperature. Similarly, for cold stress tests, seedlings were placed in a 4°C freezer; also sampled at 0, 1, 6, 12, 24, and 48 h. Concurrently, the roots, stems, leaves of the untreatment seedlings were also harvested for RNA extraction. These samples were put in liquid nitrogen and stored in −80°C. The above experiments were repeated three times to ensure precision and reproducibility.

### RNA extraction, Semi-quantitative and quantitative RT-PCR analysis

Total RNA was extracted using the RNAprep Pure Plant Kit (TIANGEN, Beijing) according to the manufacturer's instructions. Prime Script RT reagent Kit (TaKaRa, Dalian) was used for reverse transcription. The condition for Semi-quantitative PCR amplification of cDNA was as follows: denature at 94°C for 10 min and 32 (actin) or 35(gene) cycles for 94°C 30 s; 58°C (gene) or 60°C (actin) 30 s; 72°C 30 s, followed by further incubation for 5 min at 72°C (1 cycle). Furthermore, densitometry and band quantization was performed utilizing Bio-Rad Quantity One software. Quantitative PCR was performed on the Applied Biosystems 7500 real-time PCR system using Super Real PreMix Plus (SYBR Green) (TIANGEN, Beijing), as per instructions. The PCR thermal cycle conditions were as follows: denature at 95°C for 15 min and 40 cycles for 95°C, 10 s; 60°C, 20 s; 72°C, 32 s. The specificity of the PCR reactions was determined by analyzing the melting curve. The constitutive actin gene (AF288226.1) was used as the endogenous control; quantification of the 20 SiALDH gene transcript was analyzed using the 2^−ΔΔCt^ method [69]. The primers used for real-time PCR analysis were designed with primique [70] and listed in **Table S6**. The each experiment repeat three times.

### Construction of expression vector pET- *ALDH* and the expression of foxtail millet ALDH genes in response to salinity

Primers in **Table S6** were subsequently used for cloning *SiALDH* genes from cDNA. Amplification was performed as follows: 10 min at 95°C; 32 cycles of 40 s at 94°C, 2 min at 68°C; followed by a final extension for 10 min at 72°C. PET-28a (+) was linearized via BamHI and SacI restriction digestion. Then according to the In-Fusion HD Cloning System instructions, the cloned *ALDH* genes were sub-cloned into the PET-28a (+). For functional expression, E.coli Rosetta competent cells (CoWin Bioscience Co., Ltd, Beijing) were transformed with either the recombinant plasmid (PET-ALDH/Rosetta) or with PET-28a (+) vector lacking Si*ALDH* genes (Control). The PET-*ALDH*/Rosetta and control were both allowed to grow overnight with shaking at 37°C. We next transferred 400 µl of each culture mixture into a fresh 40 ml liquid LB with kanamycin and shaken at 37°C for about 3 h. β-D-thiogalactopyranoside (IPTG) was then added to a final concentration of 0.5 mmol/L in order to induce the expression of the inserted gene. After a 16 h induction (16°C), the samples (adjusting the original A600 value of all *E.coli* groups to the same value) were spotted onto the LB agar plates supplemented with NaCl (500 mmol/L, 800 mmol/L). The experiment was repeated three times with similar results.

### Data Analysis

Statistical analyses were completed by One-way ANOVA followed by Tukey's test utilizing Origin 9.1 software. *P* values of less than 0.05 were considered statistically significant.

## Supporting Information

Figure S1 **Phylogenetic analysis and exon-intron structures of foxtail millet ALDH from the same family.** Numbers above or below branches of the tree indicate bootstrap values. Coding exons, represented by ashy, were drawn to scale. Lines connecting two exons represent introns.
(TIF)

Figure S2 **The SDS-PAGE analysis for recombinant PET-ALDH.** Total proteins from SiALDH2C1, SiALDH2C2, SiALDH2B2, SiALDH10A2, SiALDH5F1, SiALDH22A1, SiALDH3E1, SiALDH3E2, SiALDH11A1, and SiALDH12A1 were separated by SDS-PAGE. The differential protein bands near the calculated molecular mass of polypeptides expressed by SiALDH2C1, SiALDH2C2, SiALDH2B2, SiALDH10A2, SiALDH5F1, SiALDH22A1, SiALDH3E1, SiALDH3E2, SiALDH11A1, and SiALDH12A1 were about 61 kDa, 64 kDa, 63 kDa, 60 kDa, 62 kDa, 62 kDa, 58 kDa, 57 kDa, 59 kDa, and 71 kDa, respectively.
(TIF)

Table S1 **Numbers of ALDH family members identified in various organisms**.
(DOCX)

Table S2 **The Ka/Ks ratios and estimated divergence time for orthologous ALDH proteins between foxtail millet and rice and paralogous ALDH proteins in foxtail millet.**
(DOCX)

Table S3 **The promoter analysis of the 20 foxtail millet ALDH genes.**
(XLSX)

Table S4 **Relative expression values of 20 foxtail millet ALDH genes in response to various stress and hormone treatments.** One-way ANOVA analysis of variance showed significant differences between group means (P<0.05). Tukey's multiple comparison test showed significant differences between the means of groups depicted by the different letters (P<0.05).
(XLSX)

Table S5 **Paralogous gene pairs within foxtail millet and orthologous gene pairs between foxtail millet and rice.**
(DOCX)

Table S6 **A list of Primers sequences of 20 foxtail millet ALDH genes for real-time PCR and gene clone.**
(DOCX)

Text S1 **The information of 20 SiALDH genes.** 20 SiALDH gene CDS and protein sequenced were completely listed.
(TXT)

Text S2 **Multiple alignments of ALDH amino acid sequences from** ***A. thaliana*** **,** ***O. sativa*** **,** ***S. Bicolor*** **,** ***Z. Mays*** **and** ***S.italica*** **.**
(TXT)
