# Supplementary figures and images for: Characteristics and Expression Patterns of the Aldehyde Dehydrogenase (ALDH) Gene Superfamily of Foxtail Millet (Setaria italica L.)
Source: PLoS One. 2014 Jul 2;9(7):e101136. doi: 10.1371/journal.pone.0101136 (PMC4079696; doi:10.1371/journal.pone.0101136)

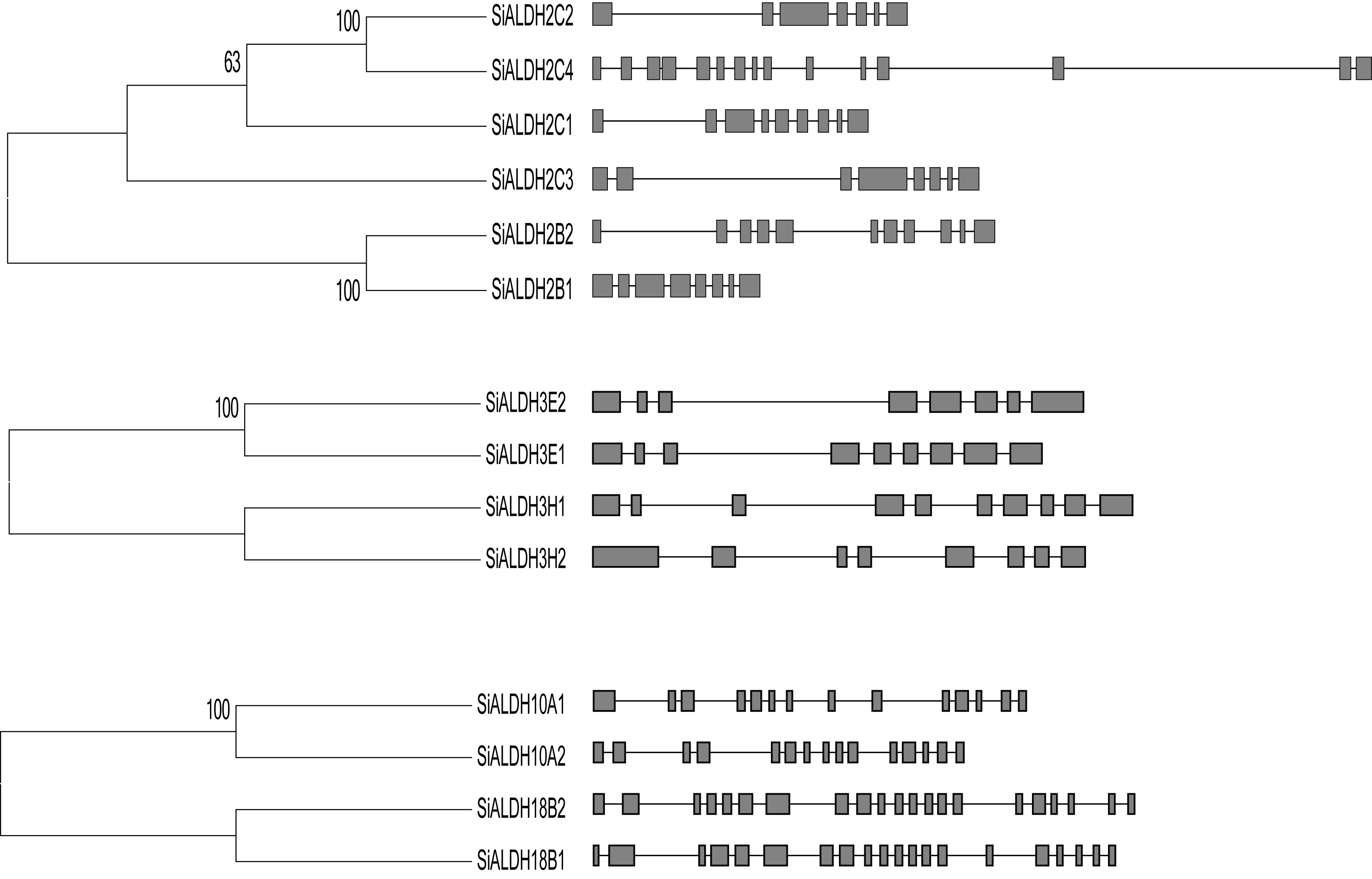

Supplement: Figure S1 — Phylogenetic analysis and exon-intron structures of foxtail millet ALDH from the same family. Numbers above or below branches of the tree indicate bootstrap values. Coding exons, represented by ashy, were drawn to scale. Lines connecting two exons represent introns. (TIF) [file pone.0101136.s001.tif]

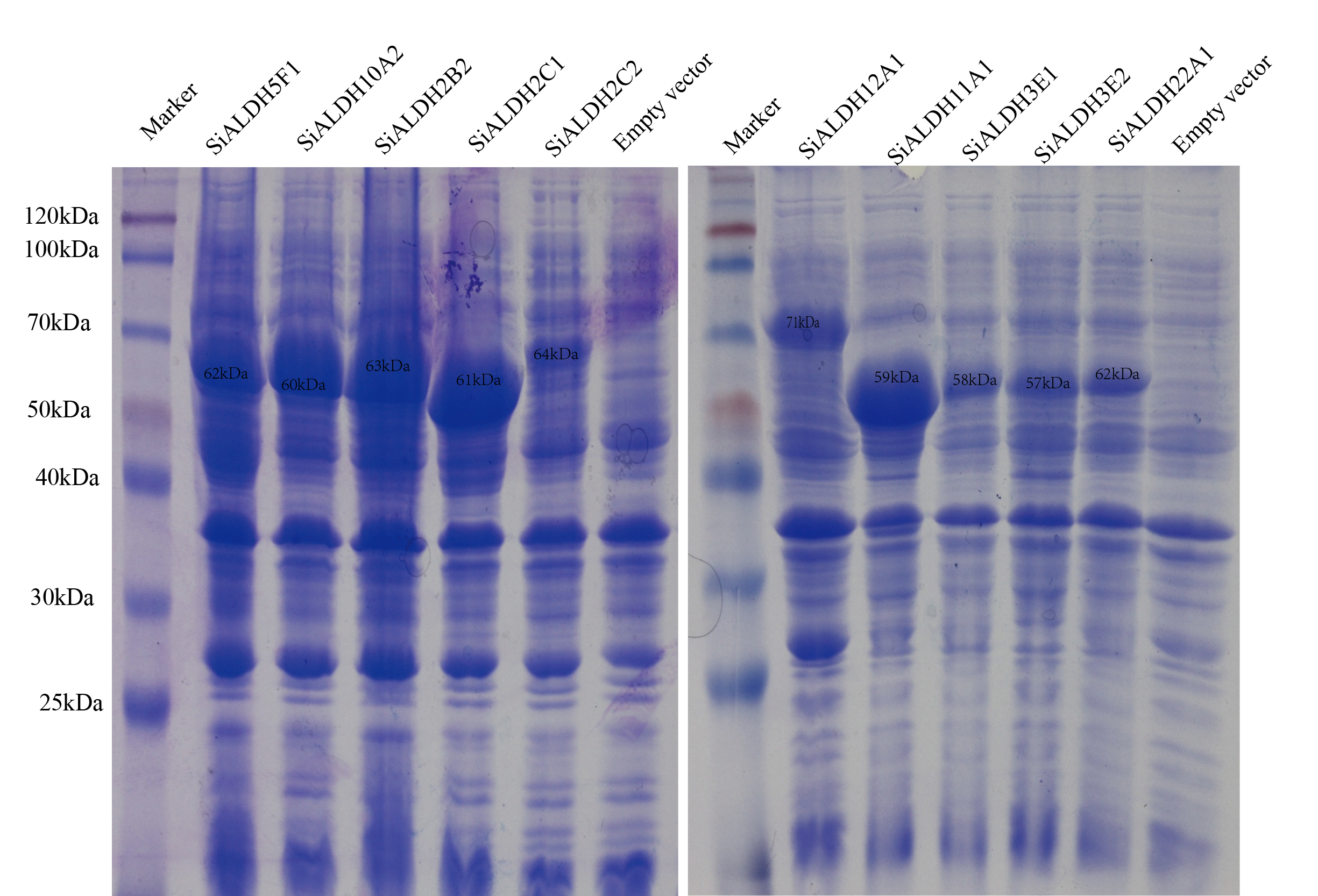

Supplement: Figure S2 — The SDS-PAGE analysis for recombinant PET-ALDH. Total proteins from SiALDH2C1, SiALDH2C2, SiALDH2B2, SiALDH10A2, SiALDH5F1, SiALDH22A1, SiALDH3E1, SiALDH3E2, SiALDH11A1, and SiALDH12A1 were separated by SDS-PAGE. The differential protein bands near the calculated molecular mass of polypeptides expressed by SiALDH2C1, SiALDH2C2, SiALDH2B2, SiALDH10A2, SiALDH5F1, SiALDH22A1, SiALDH3E1, SiALDH3E2, SiALDH11A1, and SiALDH12A1 were about 61 kDa, 64 kDa, 63 kDa, 60 kDa, 62 kDa, 62 kDa, 58 kDa, 57 kDa, 59 kDa, and 71 kDa, respectively. (TIF) [file pone.0101136.s002.tif]
