# Supplementary material for: Characteristics and Expression Patterns of the Aldehyde Dehydrogenase (ALDH) Gene Superfamily of Foxtail Millet (Setaria italica L.)
Source: PLoS One. 2014 Jul 2;9(7):e101136. doi: 10.1371/journal.pone.0101136 (PMC4079696; doi:10.1371/journal.pone.0101136)
Supplement: Table S1 — Numbers of ALDH family members identified in various organisms. (DOCX) [file pone.0101136.s003.docx]

**Table S1**. Numbers of ALDH family members identified in various organisms

| Species | ALDH Family | | | | | | | | | | | | | | | | | | | Total | | | | | |
| --- | --- | --- | --- | --- | --- | --- | --- | --- | --- | --- | --- | --- | --- | --- | --- | --- | --- | --- | --- | --- | --- | --- | --- | --- | --- |
|  | 2 | 3 | 4 | 5 | 6 | 7 | 8 | 9 | 10 | 11 | 12 | 13 | 14 | 15 | 16 | 17 | 18 | 19 | 20 | 21 | 22 | 23 | 24 |  |  |
| *Arabidopsis thaliana* | 3 | 3 | - | 1 | 1 | 1 | - | - | 2 | 1 | 1 | - | - | - | - | - | 2 | - | - | - | 1 | - | - | 16 |  |
| *Oryza sativa* | 5 | 5 | - | 1 | 1 | 1 | - | - | 2 | 1 | 1 | - | - | - | - | - | 2 | - | - | - | 1 | - | - | 20 |  |
| *Sorghum bicolor* | 5 | 4 | - | 1 | 1 | 1 | - | - | 2 | 1 | 1 | - | - | - | - | - | 2 | - | - | - | 1 | - | - | 19 |  |
| *Zea mays* | 6 | 5 | - | 2 | 1 | 1 | - | - | 2 | 1 | 1 | - | - | - | - | - | 2 | - | - | - | 1 | - | - | 22 |  |
| *Setaria italica* | 6 | 4 | - | 1 | 1 | 1 | - | - | 2 | 1 | 1 | - | - | - | - | - | 2 | - | - | - | 1 | - | - | 20 |  |
| *Chlamydomonas reinhardtii* | 1 | - | - | 1 | 1 | - | - | - | 1 | 1 | 1 | - | - | - | - | - | 1 | - | - | - | 1 | - | 1 | 9 |  |
| *Physcomitrella patens* | 2 | 5 | - | 2 | 1 | 1 | - | - | 1 | 5 | 1 | - | - | - | - | - | 1 | - | - | 1 | - | 1 | - | 20 |  |
| *Populus trichocarpa* | 4 | 6 | - | 1 | 4 | 2 | - | - | 2 | 3 | 1 | - | - | - | - | - | 2 | - | - | - | 1 | - | - | 26 |  |
| *Vitis vinifera* | 5 | 4 | - | 3 | 3 | 2 | - | - | 2 | 2 | 1 | - | - | - | - | - | 2 | - | - | - | 1 | - | - | 25 |  |
| *Glycine max* | 5 | 1 | - | - | - | 4 | - | - | 6 | 2 | - | - | - | - | - | - | - | - | - | - | - | - | - | 18 |  |
| *Volvox carteri* | 1 | - | - | - | 1 | - | - | - | 1 | 1 | 1 | - | - | - | - | - | 1 | - | - | - | 1 | - | - | 7 |  |
| *Homo sapiens* | 7 | 4 | 1 | 1 | 1 | 1 | 1 | 1 | - | - | - | - | - | - | 1 | - | 1 | - | - | - | - | - | - | 19 |  |
