# Supplementary material for: Characteristics and Expression Patterns of the Aldehyde Dehydrogenase (ALDH) Gene Superfamily of Foxtail Millet (Setaria italica L.)
Source: PLoS One. 2014 Jul 2;9(7):e101136. doi: 10.1371/journal.pone.0101136 (PMC4079696; doi:10.1371/journal.pone.0101136)
Supplement: Table S2 — The Ka/Ks ratios and estimated divergence time for orthologous ALDH proteins between foxtail millet and rice and paralogous ALDH proteins in foxtail millet. (DOCX) [file pone.0101136.s004.docx]

**Table S2.** The Ka/Ks ratios and estimated divergence time for orthologous ALDH proteins between foxtail millet and rice and paralogous ALDH proteins in foxtail millet

| Foxtail-Rice | | | | | Ks | Ka | Ka/Ks | MYA |
| --- | --- | --- | --- | --- | --- | --- | --- | --- |
| ID(NAME) | chrm | ID(NAME) | | chrm |  |  |  |  |
| Si000743m（SiALDH2C3） | 5 | LOC_Os01g40860.1  (OsALDH2C1) | | 1 | 1.5361 | 0.0677 | 0.044 | 118.16 |
| Si006183m  (SiALDH2B2) | 4 | LOC_Os02g49720.1  (OsALDH2B5) | | 2 | 2.8804 | 0.1054 | 0.037 | 221.57 |
| Si016807m  (SiALDH2B1) | 1 | LOC_Os06g15990.1  (OsALDH2B1) | | 6 | 4.3178 | 0.1254 | 0.029 | 332.14 |
| Si009984m  (SiALDH3E2) | 7 | LOC_Os04g45720.1  (OsALDH3E2) | | 4 | 1.6303 | 0.103 | 0.063 | 125.41 |
| Si009984m  (SiALDH3E2) | 7 | LOC_Os02g43194.1  (OsALDH3E1) | | 2 | 4.6986 | 0.2159 | 0.046 | 361.43 |
| Si017050m  (SiALDH3E1) | 1 | LOC_Os04g45720.1  (OsALDH3E2) | | 4 | 1.3849 | 0.1283 | 0.093 | 106.53 |
| Si017050m  (SiALDH3E1) | 1 | LOC_Os02g43194.1  (OsALDH3E1) | | 2 | 2.4544 | 0.1892 | 0.077 | 188.80 |
| Si000348m  (SiALDH18B2) | 5 | LOC_Os05g38150.1  (OsALDH18B1) | | 5 | 1.1628 | 0.1258 | 0.108 | 89.45 |
| Si021235m  (SiALDH18B1) | 3 | LOC_Os01g62900.1  (OsALDH18B2) | | 1 | 1.0281 | 0.1303 | 0.127 | 79.08 |
|  |  |  | | Average | 2.3437 | 0.1323 | 0.069 | 180.28 |
| Si016884m  (SiALDH5F1) | 1 | LOC_Os02g07760.1  (OsALDH5F1) | |  | 0.4331 | 0.0313 | 0.072 | 33.32 |
| Si029327m  (SiALDH6B1) | 2 | LOC_Os07g09060.1  (OsALDH6B1) | | 7 | 0.4071 | 0.0323 | 0.079 | 31.32 |
| Si029116m  (SiALDH7B1) | 2 | LOC_Os09g26880.1  (OsALDH7B6) | | 9 | 0.4132 | 0.0402 | 0.097 | 31.78 |
| Si009902m  (SiALDH10A2) | 7 | LOC_Os04g39020.1  (OsALDH10A5) | | 4 | 0.3481 | 0.0603 | 0.173 | 26.78 |
| Si013592m  (SiALDH10A1) | 6 | LOC_Os08g32870.1  (OsALDH10A9) | | 8 | 0.4532 | 0.0709 | 0.156 | 34.86 |
| Si013613m  (SiALDH11A1) | 6 | LOC_Os08g34210.1  (OsALDH11A3) | | 8 | 0.4022 | 0.0242 | 0.060 | 30.94 |
| Si021235m  (SiALDH18B1) | 3 | LOC_Os05g38150.1  (OsALDH18B1) | | 5 | 0.5655 | 0.0573 | 0.101 | 43.50 |
| Si006183m  (SiALDH2B2) | 5 | LOC_Os06g15990.1  (OsALDH2B1) | | 6 | 0.5515 | 0.0427 | 0.077 | 42.42 |
|  |  |  | | Average | 0.4467 | 0.0449 | 0.101 | 34.37 |
| Si006255m  (SiALDH2C1) | 4 | LOC_Os06g39230.1  (OsALDH2C3) | | 6 | 0.8862 | 0.0896 | 0.101 | 68.17 |
| Si016807m  (SiALDH2B1) | 1 | LOC_Os02g49720.1  (OsALDH2B5) | | 2 | 0.7838 | 0.0542 | 0.069 | 60.29 |
| Si009981m  (SiALDH3H1) | 7 | LOC_Os12g07810.1  (OsALDH3H1) | | 12 | 0.7485 | 0.12 | 0.160 | 57.58 |
| Si026307m  (SiALDH3H2) | 8 | LOC_Os11g08300.1  (OsALDH3H2) | | 11 | 0.6975 | 0.1166 | 0.167 | 53.65 |
| Si000348m  (SiALDH18B2) | 5 | LOC_Os01g62900.1  (OsALDH18B2) | | 1 | 0.6273 | 0.0571 | 0.091 | 48.25 |
|  |  |  | | Average | 0.7487 | 0.0875 | 0.118 | 57.59 |
| Paralogous pairs | | | Duplicate type |  |  |  |  |  |
| SiALDH2C2-SiALDH2C3 | | | Tandem |  | 88.9534 | 0.2098 | 0.0024 | 6842.57 |
| SiALDH2B2-SiALDH2B1 | | | Segmental |  | 6.1178 | 0.1422 | 0.0232 | 470.60 |
| SiALDH3H1-SiALDH3H2 | | | Segmental |  | 1.9368 | 0.1736 | 0.0897 | 148.98 |
| SiALDH3E1-SiALDH3E2 | | | Segmental |  | 2.8829 | 0.1794 | 0.062 | 221.76 |
| SiALDH18B1-SiALDH18B2 | | | Segmental |  | 1.1113 | 0.1918 | 0.1726 | 85.48 |
|  | | |  | Average | 20.200 | 0.1794 | 0.0699 | 1553.88 |
