# Supplementary material for: Characteristics and Expression Patterns of the Aldehyde Dehydrogenase (ALDH) Gene Superfamily of Foxtail Millet (Setaria italica L.)
Source: PLoS One. 2014 Jul 2;9(7):e101136. doi: 10.1371/journal.pone.0101136 (PMC4079696; doi:10.1371/journal.pone.0101136)
Supplement: Table S5 — Paralogous gene pairs within foxtail millet and orthologous gene pairs between foxtail millet and rice. (DOCX) [file pone.0101136.s007.docx]

**Table S5. Paralogous gene pairs within foxtail millet and orthologous gene pairs between foxtail millet and rice.**

| **Gene 1** | **locus** | **Chr.** | **Gene 2** | **locus** | **Chr.** | **e-value** |
| --- | --- | --- | --- | --- | --- | --- |
| SiALDH2C2 | 26987706-26991146 | 5 | SiALDH2C3 | 26969417-26974619 | 5 | 1.000000e-250 |
| SiALDH2B2 | 12210909-12216324 | 4 | SiALDH2B1 | 37023462- 37026408 | 1 | 1.000000e-250 |
| SiALDH3H1 | 33062236- 33066290 | 7 | SiALDH3H2 | 5726458-5730156 | 8 | 1.000000e-250 |
| SiALDH3E1 | 32844721-32848094 | 1 | SiALDH3E2 | 26137545-26141230 | 7 | 1.000000e-250 |
| SiALDH18B1 | 17827899-17834246 | 3 | SiALDH18B2 | 41405613-41412194 | 5 | 1.000000e-250 |
| SiALDH2C3 | 26969417-26974619 | 5 | LOC_Os01g40860.1 | 23097408-23106072 | 1 | 1.000000e-250 |
| SiALDH2C1 | 34876087-34879797 | 4 | LOC_Os06g39230.1 | 23289734-23292106 | 6 | 1.000000e-250 |
| SiALDH2B2 | 12210909-12216324 | 4 | LOC_Os06g15990.1 | 9091242-9096200 | 6 | 1.000000e-250 |
| SiALDH2B2 | 12210909-12216324 | 4 | LOC_Os02g49720.1 | 30393511-30396549 | 2 | 1.000000e-250 |
| SiALDH2B1 | 37023462-37026408 | 1 | LOC_Os02g49720.1 | 30393511-30396549 | 2 | 1.000000e-250 |
| SiALDH2B1 | 37023462-37026408 | 1 | LOC_Os06g15990.1 | 9091242-9096200 | 6 | 1.000000e-250 |
| SiALDH3H1 | 33062236-33066290 | 7 | LOC_Os12g07810.1 | 3947161-3955706 | 12 | 1.000000e-250 |
| SiALDH3H2 | 5726458-5730156 | 8 | LOC_Os11g08300.1 | 4375459-4379775 | 11 | 1.000000e-250 |
| SiALDH3E2 | 26137545-26141230 | 7 | LOC_Os04g45720.1 | 27044262-27048626 | 4 | 1.000000e-250 |
| SiALDH3E2 | 26137545-26141230 | 7 | LOC_Os02g43194.1 | 26034562-26041266 | 2 | 1.000000e-250 |
| SiALDH3E1 | 32844721-32848094 | 1 | LOC_Os02g43194.1 | 26034562-26041266 | 2 | 1.000000e-250 |
| SiALDH3E1 | 32844721-32848094 | 1 | LOC_Os04g45720.1 | 27044262-27048626 | 4 | 1.000000e-250 |
| SiALDH5F1 | 6430588-6439584 | 1 | LOC_Os02g07760.1 | 4044686-4051889 | 2 | 1.000000e-250 |
| SiALDH6B1 | 5530866-5538352 | 2 | LOC_Os07g09060.1 | 4734733-4741222 | 7 | 1.000000e-250 |
| SiALDH7B1 | 32046354-32052864 | 2 | LOC_Os09g26880.1 | 16330436-16338351 | 9 | 1.000000e-250 |
| SiALDH10A2 | 22134038-22138541 | 7 | LOC_Os04g39020.1 | 23171793-23176220 | 4 | 1.000000e-250 |
| SiALDH10A1 | 27019488-27024751 | 6 | LOC_Os08g32870.1 | 20379969-20385828 | 8 | 1.000000e-250 |
| SiALDH11A1 | 28541757-28545801 | 6 | LOC_Os08g34210.1 | 21446089-21450612 | 8 | 1.000000e-250 |
| SiALDH18B2 | 41405613-41412194 | 5 | LOC_Os01g62900.1 | 36431167-36439728 | 1 | 1.000000e-250 |
| SiALDH18B2 | 41405613-41412194 | 5 | LOC_Os05g38150.1 | 22374753-22380703 | 5 | 1.000000e-250 |
| SiALDH18B1 | 17827899-17834246 | 3 | LOC_Os01g62900.1 | 36431167-36439728 | 1 | 1.000000e-250 |
| SiALDH18B1 | 17827899-17834246 | 3 | LOC_Os05g38150.1 | 22374753-22380703 | 5 | 1.000000e-250 |

Regenerate this analysis: <http://genomevolution.org/r/cxnv>, <http://genomevolution.org/r/cxnp>
