# Supplementary material for: Characteristics and Expression Patterns of the Aldehyde Dehydrogenase (ALDH) Gene Superfamily of Foxtail Millet (Setaria italica L.)
Source: PLoS One. 2014 Jul 2;9(7):e101136. doi: 10.1371/journal.pone.0101136 (PMC4079696; doi:10.1371/journal.pone.0101136)
Supplement: Table S6 — A list of Primers sequences of 20 foxtail millet ALDH genes for real-time PCR and gene clone. (DOCX) [file pone.0101136.s008.docx]

**Table S6. A list of primers sequences of 20 foxtail millet ALDH genes for real-time PCR and gene clone**

| Primer name | gene | 5’-3’ Nucleotide sequence | Target |
| --- | --- | --- | --- |
| ALDH1-F | SiALDH2C2 | TTGAGCCCACCATCTTCGT | Real-time PCR |
| ALDH1-R | SiALDH2C2 | TAGCAGTTGACCCACACGGT | Real-time PCR |
| ALDH2-F | SiALDH2C3 | CCGTTGAGGAGGTCATTCA | Real-time PCR |
| ALDH2-R | SiALDH2C3 | CGGTCTTCGTCTGGAGATACTT | Real-time PCR |
| ALDH3-F | SiALDH2C4 | GGCAAGTCGCCACTGATAAT | Real-time PCR |
| ALDH3-R | SiALDH2C4 | GCTACACAAACCTCGCCCTT | Real-time PCR |
| ALDH4-F | SiALDH2C1 | CGAAGAGCAACCTGAAACC | Real-time PCR |
| ALDH4-R | SiALDH2C1 | TACTGAGCCTTGTCCACCTG | Real-time PCR |
| ALDH5-F | SiALDH2B2 | TGCTGAACAAACTCCTCTATCG | Real-time PCR |
| ALDH5-R | SiALDH2B2 | TTGCCTCCTAACTCCAGTGTC | Real-time PCR |
| ALDH6-F | SiALDH2B1 | GACCATCCTCAAGTTCAGCG | Real-time PCR |
| ALDH6-R | SiALDH2B1 | GTCGTAGCAGTTCACCCACA | Real-time PCR |
| ALDH7-F | SiALDH10A2 | TGTTGTCAGTGAAGGGCAGT | Real-time PCR |
| ALDH7-R | SiALDH10A2 | GTATCATTTGCGAGTTCCACAG | Real-time PCR |
| ALDH8-F | SiALDH10A1 | TCACCAGTTACCCTTCCGA | Real-time PCR |
| ALDH8-R | SiALDH10A1 | CCAGCATCAGGACCTAAACC | Real-time PCR |
| ALDH9-F | SiALDH5F1 | TCTTTGGAACTTGGTGGGA | Real-time PCR |
| ALDH9-R | SiALDH5F1 | AGGACCCTGTGATGTGCTCT | Real-time PCR |
| ALDH10-F | SiALDH22A1 | CAAGAAGAGGCATTTGGACC | Real-time PCR |
| ALDH10-R | SiALDH22A1 | TTGATTGCTGCTACACCACAG | Real-time PCR |
| ALDH11-F | SiALDH3H1 | CCAGCATCCACCTCACAAAT | Real-time PCR |
| ALDH11-R | SiALDH3H1 | CCTACAGCAGCCTGAACCAT | Real-time PCR |
| ALDH12-F | SiALDH3H2 | GCGACCCTATCTCTTGTCTGA | Real-time PCR |
| ALDH12-R | SiALDH3H2 | TTGGGAGTAGTGAAGGCGA | Real-time PCR |
| ALDH13-F | SiALDH3E2 | CGAGATGTTCAGCCACAAGA | Real-time PCR |
| ALDH13-R | SiALDH3E2 | GAGAGCACGAAGGCGAAGTA | Real-time PCR |
| ALDH14-F | SiALDH3E1 | CGAGATGTTCAGCCACAAGA | Real-time PCR |
| ALDH14-R | SiALDH3E1 | TCAGTGCGAAGAAGAGCGA | Real-time PCR |
| ALDH15-F | SiALDH11A1 | CAATCCCGCCATTCAACTAT | Real-time PCR |
| ALDH15-R | SiALDH11A1 | GACCATTCCAGCCTTCTTTG | Real-time PCR |
| ALDH16-F | SiALDH7B1 | TCTGCGGAAACTGTGTTGTC | Real-time PCR |
| ALDH16-R | SiALDH7B1 | TGAACCATTAGACCAGCCCT | Real-time PCR |
| ALDH17-F | SiALDH12A1 | AATGGGACAACATACGCTGG | Real-time PCR |
| ALDH17-R | SiALDH12A1 | AGACAAGTTTGATGGCTTCTGG | Real-time PCR |
| ALDH18-F | SiALDH6B1 | TCAGGCAAGCGTGTTCAGT | Real-time PCR |
| ALDH18-R | SiALDH6B1 | AACCCAGCAGCAATAAGGG | Real-time PCR |
| ALDH19-F | SiALDH18B2 | TGTTGTCATTGCCAGTGGAT | Real-time PCR |
| ALDH19-R | SiALDH18B2 | CGATTTCTCATAACCAGCACC | Real-time PCR |
| ALDH20-F | SiALDH18B1 | ACTGATGCCCTTCCTGACAC | Real-time PCR |
| ALDH20-R | SiALDH18B1 | GATGCCTTGATTTGCGAAAC | Real-time PCR |
| Actin-F | AF288226.1 | CATCACACCTTCTACAACGAGC | Real-time PCR |
| Actin-R | AF288226.1 | CAGAGTCCAACACGATACCTGT | Real-time PCR |
| InALDH1-F | SiALDH2C2 | ATGGGTCGCGGATCCATGCCTATTTATAAGGCGGC | Clone gene |
| InALDH1-R | SiALDH2C2 | AAGCTTGTCGACGGAGTCAGTACCACGGCGAGTC | Clone gene |
| InALDH4-F | SiALDH2C1 | ATGGGTCGCGGATCCATGGGGAGCGAGGGGAATG | Clone gene |
| InALDH4-R | SiALDH2C1 | AAGCTTGTCGACGGAGTCACAGCCAGGGCGAGTC | Clone gene |
| InALDH5-F | SiALDH2B2 | ATGGGTCGCGGATCCATGGCACGGAGGGCCGCCT | Clone gene |
| InALDH5-R | SiALDH2B2 | AAGCTTGTCGACGGAGCTACAACCAGGCGGCATTCT | Clone gene |
| InALDH7-F | SiALDH10A2 | ATGGGTCGCGGATCCATGGCCGCGCCGCCGCTGGT | Clone gene |
| InALDH7-R | SiALDH10A2 | AAGCTTGTCGACGGAGTTACAGCTTCGATGGAGGCTGG | Clone gene |
| InALDH9-F | SiALDH5F1 | ATGGGTCGCGGATCCATGGCGATGGCGATAGGGAT | Clone gene |
| InALDH9-R | SiALDH5F1 | AAGCTTGTCGACGGAGTCAGCCCATGTTGCCCATG | Clone gene |
| InALDH10-F | SiALDH22A1 | [ATGGGTCGCGGATCCATGAAATACTTGGGGTATTT](mailto:sunbio@sunbiotech.com.cn) | Clone gene |
| InALDH10-R | SiALDH22A1 | AAGCTTGTCGACGGAGTCATCGCCTTTTCTTCGTTG | Clone gene |
| InALDH13-F | SiALDH3E2 | ATGGGTCGCGGATCCATGGGCACCGAGGCCGGG | Clone gene |
| InALDH13-R | SiALDH3E2 | AAGCTTGTCGACGGAGTCATCTCCTGAGGCCGAGGA | Clone gene |
| InALDH14-F | SiALDH3E1 | ATGGGTCGCGGATCCATGGGGAGCGTGCCGGCGG | Clone gene |
| InALDH14-R | SiALDH3E1 | AAGCTTGTCGACGGAGTCACCTCCTCAGGCCGATC | Clone gene |
| InALDH15-F | SiALDH11A1 | ATGGGTCGCGGATCCATGGCGCTGGCGGGGACG | Clone gene |
| InALDH15-R | SiALDH11A1 | AAGCTTGTCGACGGAGTCAGCCCATGGTGTAGGATG | Clone gene |
| InALDH17-F | SiALDH12A1 | ATGGGTCGCGGATCCATGCAGAGGCCCCACAG | Clone gene |
| InALDH17-R | SiALDH12A1 | AAGCTTGTCGACGGAGTCATGTCGCAGAGGGGAGCG | Clone gene |
